# Irrigation and Crop Load Management Lessen Rain-Induced Cherry Cracking

**DOI:** 10.3390/plants11233249

**Published:** 2022-11-26

**Authors:** Victor Blanco, Pedro J. Blaya-Ros, Roque Torres-Sánchez, Rafael Domingo

**Affiliations:** 1 Department of Horticulture, Washington State University, Pullman, WA 99164, USA; 2 Departamento de Ingeniería Agronómica, Universidad Politécnica de Cartagena (UPCT), Paseo Alfonso XIII, 48, E30203 Cartagena, Spain; 3 Departamento de Automática, Ingeniería Eléctrica y Tecnología Electrónica, Universidad Politécnica de Cartagena (UPCT), Campus de la Muralla s/n, E30202 Cartagena, Spain

**Keywords:** deficit irrigation, fruit and stem water potential, fruit quality, osmotic water potential, tree water status, thinning

## Abstract

The combined effects of deficit irrigation and crop load level on sweet cherry (*Prunus avium* L.) physiological and agronomic response were evaluated during the 2019 season in a commercial orchard located in southeastern Spain. Two irrigation treatments were imposed: (i) control treatment (CTL) irrigated above crop water requirements at 110% of crop evapotranspiration (ET_C_) and (ii) a deficit irrigation treatment (DI) irrigated at 70% ET_C_. Within each irrigation treatment, crop load was adjusted to three levels: 100% (natural crop load—high), 66% (medium crop load), and 33% (low crop load). The water relations results were more affected by the irrigation strategies applied than by the crop load management. The deficit irrigation strategy applied reduced soil water availability for DI trees, which led to a continuous decrease in their gas exchange and stem water potential. At harvest, the fruit water potential and osmotic potential of cherries from the DI treatment resulted in significantly lower values than those measured in cherries from CTL trees. On the other hand, both the irrigation strategies imposed and the crop load management used impacted fruit quality. Trees with the lowest level of crop load had fruits of greater size, regardless of the irrigation treatment assayed, and in the DI treatment, cherries from the trees with the lowest crop load were darker and more acidic than those from the trees with the highest crop load. Our results emphasize the different effects that rainfall before harvest has on mature cherries. Thus, cracked cherries at harvest represented 27.1% of the total yield of CTL trees while they were 8.3% of the total yield in DI trees. Cherries from CTL trees also showed a greater cracking index than those from DI trees. Moreover, a linear relationship between crop load and fruit cracked at harvest was observed, particularly for the CTL treatment; thus, the lower the crop load, the greater the proportion of cracked cherries.

## 1. Introduction

Cracking in sweet cherries (*Prunus avium* L.) has been described as a main physiological problem for all cultivars and a factor limiting the further increase of cherry production in several regions where rain occurs during the harvest period [1]. Moreover, climate change is likely to worsen this situation, as water scarcity will be paired with extreme episodes of heavy storms that are expected to intensify in frequency. This situation may increase the chances of having rain and hailstorms during the weeks prior to harvest and subsequently cause significant economic losses to cherry growers, which threatens commercial production in vulnerable regions. Cracked cherries are not marketable because of their poor quality (which implies short storage and shelf life) and are associated with postharvest diseases (such as decay [2]).

Fruit cracking, although not completely elucidated, is described as a result of increasing turgor pressure, which provokes the bursting of the flesh cells (“Zipper model”). It is caused by water uptake (osmotically) first for the cells of the skin and then for those in the flesh, and it happens during or right after a rainfall episode when the cherries are in the final stage of fruit development. The main route of water uptake appears to be through the fruit surface, through microcracks in the cuticle [3].

The flow of water from the soil to the fruit in cherry trees is influenced by changes in the gradients between roots, stem, leaves, and fruit water potentials, and the environmental conditions such as vapor pressure deficit, as well as the developmental stage of the fruit. A relationship was observed by Measham et al. [4] between the incidence of rainfall and the direction of water flow (sap) to the fruits through the tree. On the other hand, a larger water absorption is thought to occur through the sweet cherry’s fruit surface when the fruit is wet, especially during and after rainfall [5]. However, although related, fruit cracking incidence in an orchard is not exclusively related to the volume of rainfall or to the duration of the rainfall episode [6]. Several factors apart from the quantity and distribution of rainfall during the ripening season [4] have been related to fruit cracking, such as genetically determined susceptibility of species and cultivars [7] and the soil type and soil moisture condition of the orchard [8]. Regarding the last point, there are different approaches; on the one hand, Measham et al. [9] suggest that avoiding water stress at late stages of fruit growth can improve resistance to cracking. On the other hand, Edin et al. [10] reported that low values of soil water content might help to decrease root water uptake and fruit cracking. Other authors highlight a relationship between major changes in soil water content prior to harvest and the development of cuticular fractures in the fruit, which might cause cherry cracking [11]. In other fruit crops, water deficit and irrigation strategies that involve water stress management in different phenological periods (such as regulated deficit irrigation (RDI)) have shown mixed effects on fruit cracking. On the one hand, Galindo et al. [12] and Goodwin et al. [13] reported in pomegranates and apples, respectively, that water stress increases the number of cracked fruits. On the other hand, Yan et al. [14] reported in plums that the cracking incidence was lower when the trees were under deficit irrigation. In cherries, tree management practices with the main purpose of reducing fruit cracking incidence have been widely studied. Thus, the use of physical barriers [15,16] or hydrophobic films [17,18] to reduce fruit wetting in the final stage of ripening or to modify fruit osmotic potential during the occurrence of rain [19] have successfully decreased cracking incidence, although they have also affected fruit quality. However, other management practices such as irrigation management might also play a key role in cracking incidence, particularly when combined with cultivation practices such as thinning, which increases fruit size and the concentration of soluble solids and consequently modifies the fruit’s osmotic and turgor potentials. Thus, this study was conducted to assess the impact of deficit irrigation and crop load management on the tree physiological response, fruit quality, and cracking incidence in sweet cherries.

## 2. Results

### 2.1. Soil-Plant-Atmosphere Water Status

**Figure 1:**
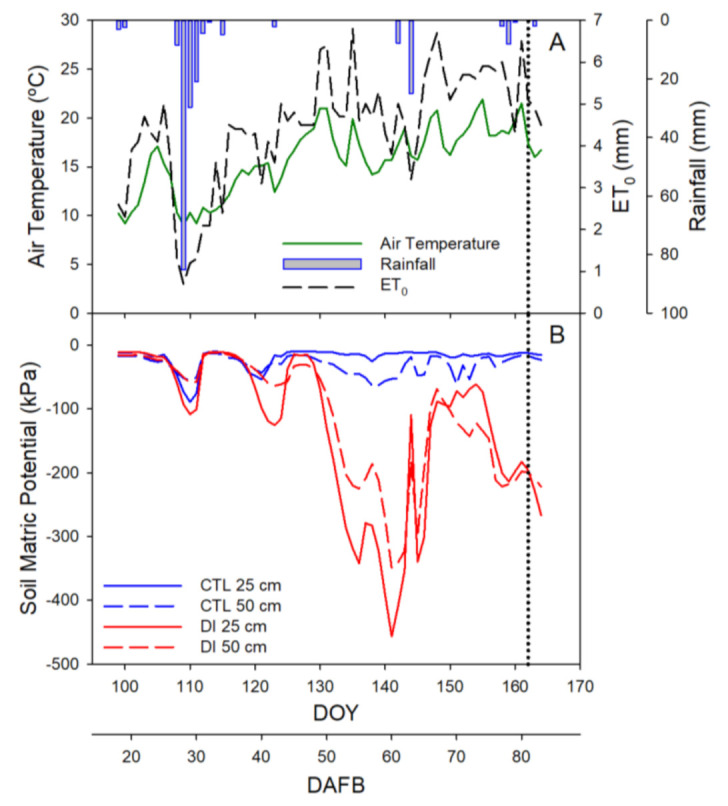
Seasonal evolution of environmental conditions (**A**): daily air temperature (green solid line), reference evapotranspiration (black dashed line) and rainfall (blue bars), and soil matric potential (**B**) at 0.25 m (solid line) and 0.50 m (dash line) depth for the two irrigation treatments, CTL (full irrigated treatment; blue) and DI (deficit irrigation treatment; red). The vertical dotted line indicates the harvest day (day of year, DOY 162; days after full bloom, 81 DAFB). Full bloom: 22 March 2019; harvest day: 11 June 2019.

Environmental conditions during the experiment varied greatly, with values that increased from air temperature and reference crop evapotranspiration (ET_0_) close to 10 °C and 2 mm d^−1^ at the beginning of the experiment to 22 °C and 6.5 mm d^−1^ at harvest, respectively. The accumulated amount of rainfall during the experiment was 206 mm, with the greatest rain episode in late April (149 mm in 5 days). However, it must be highlighted that two rain episodes happened in the three weeks prior to harvest, mid-May (35 mm) and early June (11 mm), the period when cherries are more likely to crack (Figure 1A). The accumulated ET_0_ during the experiment (the 2019 preharvest) was 311 mm and the irrigation water applied during the same period was 2031 and 1250 m^3^ ha^−1^ for the control (CTL) and deficit irrigated (DI) trees, respectively.

The soil matric potential (Ψ_m_) values clearly identified the irrigation strategies assayed. As expected, the Ψ_m_ values for CTL trees were more stable than those for DI trees and higher than −90 kPa at both depths (25 and 50 cm). The soil water availability for DI trees showed a decreasing pattern during the experiment, with minimum values that fell below −400 kPa at 25 cm depth and −300 kPa at 50 cm depth (Figure 1B). Increments in Ψ_m_ for the DI treatment were caused by the rain and the environmental variability, which led to mismatches between the tree water requirements calculated according to the previous days’ environmental conditions and the actual water needs of the trees for that period.

**Figure 2:**
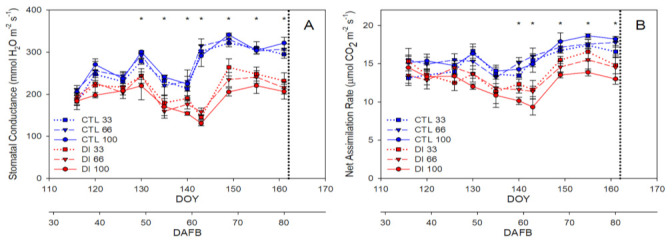
Seasonal evolution of stomatal conductance (**A**) and net assimilation rate (**B**) for the two irrigation treatments: CTL (full irrigated treatment; blue) and DI (deficit irrigation treatment; red), and the three levels of crop load: low (33%, squares), medium (66%, triangle), and high (100%, circle). Each point is the mean ± SD of three trees. Asterisks indicate significant differences between CTL and DI, according to ANOVA (*p* < 0.05). The vertical dotted line indicates the harvest day (day of year, DOY 162; days after full bloom, 81 DAFB). Full bloom: 22 March 2019; harvest day: 11 June 2019.

Reductions in soil water availability for DI trees resulted in a decrease in gas exchange. So, while during the first stage of fruit growth there were no significant differences between trees from the two irrigation treatments, after the Ψ_m_ dropped to values below −200 kPa, significant reductions in the stomatal conductance (g_s_) and net assimilation rate (P_n_) values of DI trees were detected until the end of the experiment. The mean g_s_ and P_n_ values for the last part of fruit development (stage III) for the CTL trees were similar to 320 mmol m^−2^ s^−1^ and 17 µmol m^−2^ s^−1^, with the highest values present for the trees with the highest crop load. Meanwhile, for DI trees for the same period of time, the g_s_ and P_n_ values were 200 mmol m^−2^ s^−1^ and 13 µmol m^−2^ s^−1^, respectively, with the highest values present for the trees with the lowest crop load (Figure 2).

**Figure 3:**
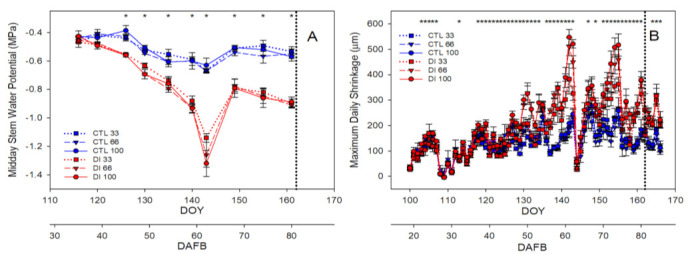
Seasonal evolution of midday stem water potential (**A**) and maximum daily branch shrinkage (**B**) for the two irrigation treatments: CTL (full irrigated treatment; blue) and DI (deficit irrigation treatment; red), and the three levels of crop load: low (33%, squares), medium (66%, triangle), and high (100%, circle). Each point is the mean ± SD of three trees. Asterisks indicate significant differences between CTL and DI, according to ANOVA (*p* < 0.05). The vertical dotted line indicates the harvest day (day of year, DOY 162; days after full bloom, 81 DAFB). Full bloom: 22 March 2019; harvest day: 11 June 2019.

Midday stem water potential (Ψ_stem_) identified the water deficit applied to the DI trees earlier than g_s_ and P_n_, and consistently showed significant differences between the two irrigation treatments imposed from DOY 126 onwards (45 DAFB; Figure 3A). Moreover, a trend in the deficit irrigation treatment was observed, particularly in those measurements below −0.90 MPa, that the trees with the lowest crop load presented higher values of stem water potential (Figure 3A). On the other hand, no clear effect of the crop load on the water status of the CTL trees was found.

The maximum daily shrinkage (MDS) was also affected by the irrigation treatments assayed and at a lower level by crop load, with values twice as high (500 µm) for those trees with a high crop load and that were deficit irrigated than those irrigated as CTL (240 µm; Figure 3B). Both indicators, the Ψ_stem_ and the MDS, were able to identify the changes produced by the 30 mm rainfall (144 DOY; 63 DAFB) in the tree water status of the DI trees. Thus, Ψ_stem_ values gradually decreased as MDS values increased in DI trees throughout the experiment until the day prior to the rainfall, with Ψ_stem_ and MDS values similar to −1.15 MPa and 350 µm for those trees with a low crop load and −1.30 MPa and 500 µm for those with a medium and high crop load. After the rain episode, the MDS values of the DI trees fell below those recorded in the CTL trees for three days, and even one week after the rain, Ψ_stem_ mean values for all DI trees were similar to −0.80 MPa (Figure 3).

### 2.2. Fruit Water Relations

**Figure 4:**
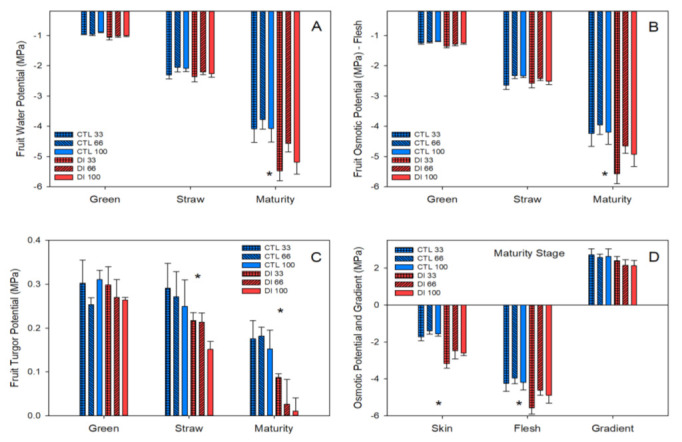
Fruit water potential (**A**), fruit osmotic potential (**B**), fruit turgor potential (**C**), and fruit osmotic potential gradient between skin and flesh at harvest (**D**) for the two irrigation treatments: CTL (full irrigated treatment; blue) and DI (deficit irrigation treatment; red), and the three levels of crop load: low (33%), medium (66%), and high (100%). Each bar is the mean ± SD of six fruits. Asterisks indicate significant differences between CTL and DI, according to ANOVA (*p* < 0.05).

The fruit water potential (Ψ_fruit_) values of both CTL and DI trees were always lower than the stem water potential values measured in the corresponding tree. Moreover, unlike Ψ_stem_, Ψ_fruit_ did not distinguish between irrigation treatments until the fruit reached maturity, when the fruit from DI trees had lower values than that from CTL trees, −5.0 and − 4.1 MPa, respectively; no trend regarding crop load was observed in any of the irrigation treatments assayed (Figure 4A). As expected, fruit osmotic potential (Ψ_πf_) was strongly related to Ψ_fruit_ with mean values that, at the green, straw and maturity stages, were lower by 29, 12, and 2%, respectively, than those of Ψ_fruit_ (Figure 4B). Both Ψ_fruit_ and Ψ_πf_ only showed significant differences between irrigation treatments at the maturity stage (Figure 4A,B). 

The estimated fruit turgor potential (Ψ_ρ_) showed a different evolution pattern than Ψ_πf_ and Ψ_fruit_. It was the highest at the fruit green stage and, while it was stable at the straw stage for CTL fruits (with values of 0.28 MPa), it decreased to values that were close to 0.20 MPa for fruits from DI treatment; moreover, in the DI treatment, it was noted that the fruit from the trees with the highest crop load was that with the lowest values of Ψ_ρ_ (Figure 4C). These differences continued and increased at the fruit maturity stage when a trend related to the crop load was found for the Ψ_ρ_ values of DI fruits. Thus, when the Ψ_ρ_ for the fruit of the CTL and DI treatments was compared, we found that for the fruit from trees with low, medium, and high crop load, Ψ_ρ_ of the fruit from CTL trees was twice, seven, and twelve times higher, respectively, than that of the fruit from DI trees. It must be highlighted that, although the mean value of the estimated Ψ_ρ_ of all treatments, irrigation, and crop load resulted in values above zero at the maturity stage, the estimated Ψ_ρ_ in some fruits from trees with high and medium crop load and deficit irrigation was negative as a consequence of the water deficit and the crop load.

At fruit maturity, the osmotic potential of the skin of the fruit (Ψ_πs_) was measured and compared with that of the flesh. A similar trend was detected with significant differences between irrigation treatments but with higher values than those reported by Ψ_πf_ (Figure 4). The skin of the cherries from the CTL trees had values of osmotic potential (Ψ_πs_ = −1.6 MPa) that were 38% of that reported by the flesh (Ψ_πf_ = −4.1 MPa), while the DI cherries had values of Ψ_πs_ (−2.8 MPa) that were 56% of that reported by the Ψ_πf_ (−5.0 MPa). When the gradients of the osmotic potential between skin and flesh were compared, no differences were detected, neither between irrigation treatments nor between crop loads, with values that ranged between 2.1 and 2.6 MPa. However, it was observed that the differences between the skin and flesh osmotic potentials showed a trend toward greater values in cherries from CTL compared to DI (Figure 4D).

### 2.3. Fruit Yield and Quality

The average trunk cross-sectional area (TCSA) of the trees considered in this study ranged from 289 to 336 cm^2^, without significant differences among the different crop levels. Tree crop load was adjusted to three levels (low, medium, and high), which corresponded to 2.4, 5.7, and 8.2 fruit cm^−2^ TCSA, respectively (the mean values of both irrigation treatments, Table 1). The fruit yield and the average number of fruits produced per tree increased along with the crop load, with mean values relevant to each crop level of 8.5, 19.1, and 26.4 kg tree^−1^ and 640, 1548, and 2148 cherries per tree, respectively (the mean values of both irrigation treatments, Table 1). The occurrence of double fruit, which was below 1.5% in all the treatments, was not influenced by the crop load. In the same vein, the proportion of cracked fruit at harvest and the cracking index were not significantly influenced by the crop load. However, in the CTL treatment, trees with low crop loads had a higher proportion of cracked fruit than trees with the natural crop load.

When the fruit yield between both irrigation treatments was compared, considering all the crop levels, there were no significant differences between both irrigation strategies regarding fruit yield, which was similar to 18 kg tree^−1^. However, significant differences emerged between irrigation treatments when the proportion of cracked fruit was compared. CTL trees had three times more cracked fruit (27.1%) than DI trees (8.3%). In a lower proportion, this trend was also observed in the determination of the cracking index carried out in the lab. As expected, the water productivity of those trees irrigated with the DI strategy was significantly higher than that of those irrigated with the CTL strategy (Table 1).

Regarding fruit quality, it was noticed that the unitary size was the most sensitive quality parameter to crop load adjustment. In both irrigation treatments, fruit equatorial diameter increased with decreasing crop load. Moreover, crop load had a different effect on fruit depending on the irrigation strategy. Thus, the quality of the fruit produced by those trees irrigated as CTL did not significantly differ among crop load levels (except for the equatorial diameter, which resulted in the lowest value for the cherries from the trees with the highest crop load). On the other hand, fruit quality was more affected by the crop load in the DI treatment. DI cherries with the lowest crop load had a similar size as those from the CTL treatment (31 mm of diameter and 13 g of unitary mass) and were darker and more acidic than those from the highest crop load within the same irrigation treatment (Table 2).

**Figure 5:**
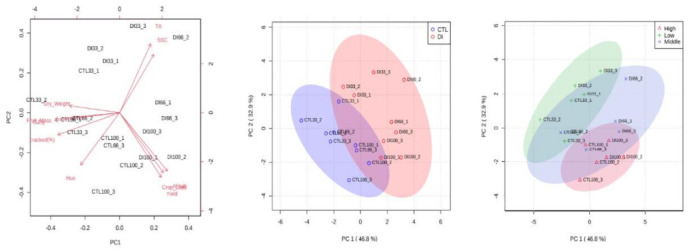
Principal component analysis (PCA) biplot of the fruit quality characteristics, expressed as vectors, of fruit from the two irrigation treatments, CTL (full irrigated treatment) and DI (deficit irrigation treatment), and the three levels of crop load: low (33%), medium (66%), and high (100%). The two principal components of the PCA explained 79.7% of the variation in the measured data.

There were no significant differences in fruit fresh mass between the medium and high crop load levels, but it was significantly increased by 11% in the level with the lowest crop load (11.8 g vs. 13.1 g, respectively). The irrigation treatments assayed had a greater impact on the fruit quality than did the crop load (Figure 5). Thus, when the PCA was performed on the fruit quality parameters measured, the data could be classified by the irrigation treatment imposed easier than by the crop load. The PCA explained 80% of the variability of the data. Fruit quality parameters such as fruit unitary mass and equatorial diameter were located on the side of the CTL treatment and the lowest crop load level, while soluble solids concentration (SSC) was on the DI side, indicating that fruit from CTL trees had a greater size than those cherries from DI trees, which were sweeter.

### 2.4. Cracking Susceptibility

**Figure 6:**
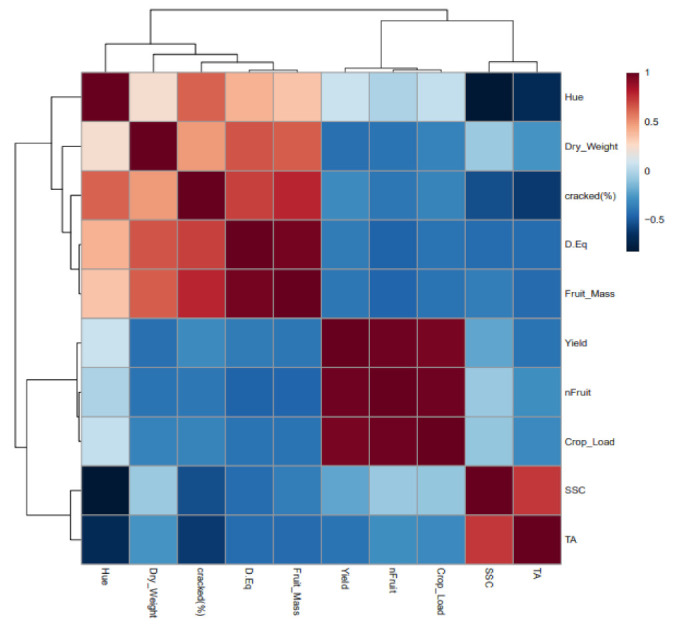
Correlation heat map of the quality parameters analyzed.

The relationship between the cracked fruit and the different physiological and quality values measured was calculated. The proportion of cracked cherries was positively related to the unitary mass of the cherry but negatively related to the titratable acidity (TA) (Figure 6). When the proportion of cracked cherries at harvest was compared with the relative water content and the osmotic water potential gradient, no relationships were found.

**Figure 7:**
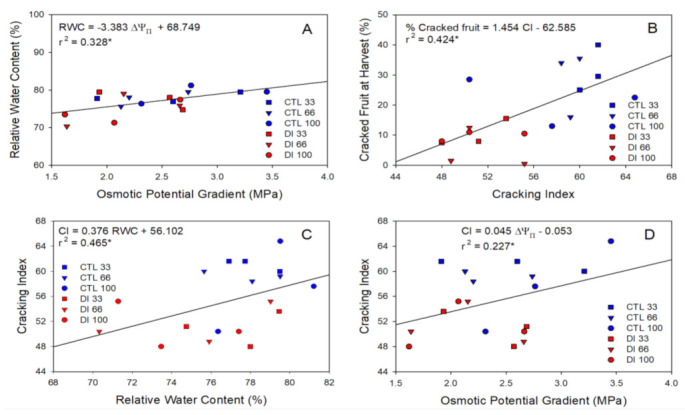
Linear regression between the fruit osmotic potential gradient (∆Ψπ) and the fruit relative water content (RWC) (**A**), the cracking index (CI) assessed in the laboratory and the proportion of fruit cracked at harvest (**B**), the fruit relative water content and the cracking index (**C**), and the osmotic potential gradient and the cracking index (**D**) for the two irrigation treatments: CTL (full irrigated treatment; blue) and DI (deficit irrigation treatment; red), and the three levels of crop load: low (33% square), medium (66% triangle), and high (100% circle). Each point represents one replicate (tree). * indicates a significant relationship between the studied variables at *p* < 0.05.

On the other hand, the cracking index was significantly related to both parameters, particularly the relative water content (R^2^ = 0.47). When the proportion of cracked fruit at harvest per block and the cracking index measured in the laboratory were compared, a significant and positive relationship was obtained. Thus, those blocks with more than 25% cracked fruit at harvest had a cracking index value higher than 60 (Figure 7).

**Figure 8:**
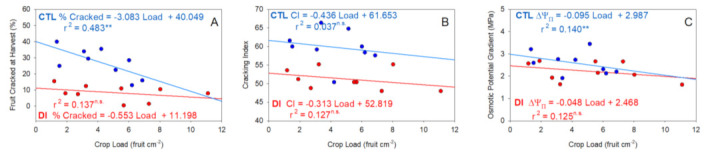
Linear regression between crop load and fruit cracked at harvest (**A**), cracking index (**B**), and osmotic potential gradient (**C**) for the two irrigation treatments: CTL (full irrigated treatment; blue) and DI (deficit irrigation treatment; red). ** indicates a significant relationship between the studied variables at *p* < 0.01 and n.s. indicates no significant relationship (*p* > 0.05).

Within each irrigation treatment, a negative linear relationship was found between crop load and both the proportion of cracked fruits at harvest and the cracking index (Figure 8). Trees with lower crop loads showed higher levels of cracking, which highlights the influence of the crop load on the trend of the fruits to crack. The trend toward a higher proportion of cracked cherries at harvest for those trees with a light crop load (<four fruit cm^−2^ TCSA) was stronger for CTL trees compared with DI trees. When both linear regression lines were compared, the linear regression line of CTL trees had an intercept and slope four and six times higher, respectively, than those of DI trees.

## 3. Discussion

The plant water status indicators considered in this study clearly identified the two irrigation strategies assayed. Among them, MDS was the indicator that first detected early water stress, followed closely by Ψ_stem_ and distantly by g_s_ and P_n_. However, when both MDS and Ψ_stem_ were compared, it was observed that MDS was more affected by the different environmental conditions (particularly by the rainfall) than Ψ_stem_. The relationships between soil and plant water status indicators in sweet cherry trees were widely studied and reported in Blanco et al. [20]. In relation to the crop load, the tree water status was more affected by the crop load level in those trees under deficit irrigation than in those irrigated to fulfill their water requirements. Likewise, Conejero et al. [21] reported in well-watered peach trees that different crop loads did not affect tree water status. In our experiment, the plant water status indicators identified that those trees with the highest crop load were under stronger water deficit conditions than those with a low crop load. Similarly, Intrigliolo and Castel [22] reported that the MDS was more affected by the crop load in the deficit irrigation treatment than in the control treatment.

Regarding the fruit water indicators, Ψ_fruit_ was not as sensitive as Ψ_stem_ and could not distinguish between irrigation treatments until harvest. In a previous experiment on sweet cherry trees [23], Ψ_fruit_ did not show significant differences between CTL trees, trees irrigated at 110% ET_C_, and trees under a slight water deficit (90% ET_C_). However, in this study, as the water deficit applied to the DI trees was more severe (70% ET_C_), Ψ_fruit_ exhibited significant differences between irrigation treatments at harvest. Ψ_fruit_ has been described as a reliable tree water status indicator for fruit trees such as pomegranate [12] and medium–late maturing peach [24]. For sweet cherry trees, as fruit development lasts for a shorter period of time, Ψ_fruit_ can only be considered a reliable water stress indicator if the deficit irrigation is moderate or severe and is applied during the last part of stage III of fruit growth. The effect of crop load on Ψ_fruit_ in peach trees was discussed by McFadyen et al. [25], who reported significant differences in the Ψ_fruit_ of fruits from trees with different levels of crop load, with more negative values in those trees with a high crop load level. In our study, Ψ_fruit_ was not significantly affected by the different levels of crop load assayed, neither in fruits from CTL trees nor in fruits from DI trees, despite the differences in fruit mass and SSC. However, a trend was detected in the DI treatment with the lowest values of Ψ_fruit_ in the cherries from the trees with the lowest level of crop load. We hypothesized that this trend to low values would be caused by the combination of the effect of the lowest level of crop load and the deficit irrigation. Mpelasoka et al. [26] reported that ‘Braeburn’ apples from trees under deficit irrigation ripened earlier than those from trees that were irrigated to satisfy their water requirements. Furthermore, for the same apple cultivar, Kelner et al. [27] reported that a low level of crop load hastened fruit maturity. In that sense, the water stress applied to DI trees promoted a slightly faster development of the cherries by increasing the soluble solids concentration, decreasing the Ψ_πf_, and finally decreasing Ψ_fruit_, which was more noticeable in the fruit from the low level of crop load. As expected, Ψ_πf_ was strongly related to Ψ_fruit_, with values slightly more negative than those reported by Moing et al. [28] in the sweet cherry cracking resistant cultivar ‘Fermina’ but following the same trend, with values that decrease as the fruit develops and the SSC increases. Winkler et al. [29] reported that, in sweet cherries, the content of the soluble sugars (glucose, fructose, and sorbitol) determines Ψ_πf_, as it represents 86% of the total osmolarity, while the organic acids and the minerals represent 7% each. Regarding the estimated turgor potential (Ψ_ρ_), cherries from the DI treatment had lower Ψ_ρ_ values than those from the CTL treatment from the straw stage to maturity. Significant lower Ψ_ρ_ values during stage III of fruit development indicated that the lower fruit size of DI fruit was more related to the effect of water deficit on the cell enlargement stage than on the first stage of cell division, as cell enlargement requires turgor to allow fruit growth [30]. The sharp fall in Ψ_ρ_ values for the mature DI cherries from trees with medium and high crop load levels showed a fruit turgor loss, which emphasized the inability of sweet cherry trees to cope with or tolerate water stress. This is in line with the slow response of the g_s_, as it took more than a month since the water deficit was applied to induce a significant stomata closure in order to reduce water losses and postpone water deficit effects. These results agree with Blaya-Ros et al. [31], who described the extreme anisohydric behavior of cherry trees. When the osmotic potential of the flesh and the skin were compared, the values measured in the flesh were more negative than those measured in the skin. For both osmotic potentials, DI fruits had significantly lower values than CTL fruits. Grimm and Knoche [32] reported that the Ψ_π_ of the flesh in sweet cherries is markedly more negative than that of the skin. Moreover, the same authors stated that the more negative the osmotic potential in the flesh, the more extensible and less likely to crack the skin will be, as a result of dehydration of the skin caused by the flesh. Consequently, large osmotic potential gradients between the skin and the flesh might cause the skin to not act as a buffer to allow the water uptake into the flesh and can trigger fruit microcracks. In this sense, the fruit from CTL trees did not result in significant differences compared to the fruit from DI trees; however, a trend toward a larger skin–flesh osmotic potential gradient was noticed in CTL fruits, which might be related to the greater cracking incidence measured in the CTL treatment at harvest. These results are in line with those reported by Correia et al. [33], who stated that irrigation techniques and strategies that decrease root water uptake have a positive impact on decreasing cracking incidence. 

Cracking incidence was positively related to the fruit mass and negatively related to SSC and TA (principal components of the Ψ_πf_, [29]). Furthermore, a negative relationship between the cracked fruit and the crop load was obtained, which implies that the fruit from both irrigation treatments with the lowest level of crop load was the most susceptible to cracking. Measham et al. [34] reported a negative relationship between the cracking incidence and the crop load in the ‘Simone’ sweet cherry; however, the relationship widely varied over multiple years. The cracking index measured in the lab was significantly related to the results of cracked fruit at harvest. Both measures clearly identify that cherries from the CTL treatment were more prompt to crack than those from the DI treatment. Another complementary mechanism that should be taken into consideration to explain the significantly lower susceptibility to crack of fruit from trees under water deficit is the modification of the cuticular wax. Several authors have highlighted that fruits from plants under drought stress increase cuticle wax load as a mechanism to reduce transpiration rates [35,36]. The larger amount of wax reported in fruits from deficit irrigated trees compared to that naturally present in those fruits from trees under no water restrictions might have played a role as a thick physical barrier that protects the fruit by keeping the rainwater from directly contacting the skin of the fruit.

As with cracking, fruit quality was also affected by both water deficit and crop load. The fruit characteristics of both CTL and DI trees at harvest were consistent with those values reported as optimal for ‘Prime Giant’ sweet cherries, with unitary mass values higher than 10 g, a mahogany color, and more than 17% SSC [37]. Similar results, increments of the unitary mass, and the SSC values of the cherries from trees with low crop load have been previously reported in the sweet cherry [38,39]. On the other hand, a decrease in the fruit unitary mass has been reported in sweet cherries as a result of a high fruit yield or a low leaf-to-fruit ratio [40,41]. The combination of a low or medium crop load and deficit irrigation did not negatively affect fruit size, color, SSC, or TA, and furthermore, it was not as affected by cracking at harvest as CTL fruit was. Thus, both combinations stand out as good options for growers facing water scarcity while producing high-quality fruit. 

## 4. Materials and Methods

### 4.1. Experimental Site, Plant Material and Treatments

The study was conducted in a 0.5 ha commercial orchard located in Jumilla (Murcia, Spain, 38°8’ N; 1°22’ W) during the 2019 growing season. The climate is typically Mediterranean, with low rainfall distributed in autumn and spring, hot dry summers, and mild winters. The average annual reference evapotranspiration (ET_0_) for the period from 2015 to 2018 was 1219 mm, and the average annual rainfall was 313 mm. The soil has a sandy loam texture and is moderately stony, with 0.32 meq 100 g^−1^ potassium, 108.67 mg kg^−1^ available phosphorus, and 2.7% active limestone. The irrigation water had 0.8 dS m^−1^ of electrical conductivity.

The plant material consisted of nineteen-year-old ‘Prime Giant’ sweet cherry trees grafted on ‘SL64′ rootstock, and ‘Early Lory’ and ‘Brooks’ used as pollinizers, spaced at 5 m × 3 m. Trees were drip-irrigated using a single drip line for each tree row, with three pressure-compensated emitters per tree, each with a discharge rate of 4 L h^−1^.

The different irrigation treatments were initiated each season before flowering and suspended at the end of November. Full bloom was on 22 March (day of year (DOY) 81) and fruit was harvested on 11 June (DOY 162: 81 days after full bloom (DAFB)). The horticultural practices used (e.g., fertilization, weed control, and pruning) were the same for the trees of all treatments. Fertilization was applied through the irrigation system with the water and was the same in all treatments regardless of the amount of water applied. The fertilization program consisted of 63 kg ha^−1^ of N, 30 kg ha^−1^ of P_2_O_5_, 107 kg ha^−1^ of K_2_O, and 8 kg ha^−1^ of CaO.

Two irrigation treatments were applied: a control (CTL) irrigated at 110% of crop evapotranspiration (ET_C_) to maintain non-limiting soil water conditions and a deficit irrigation treatment (DI) irrigated at 70% of ET_C_. Within each irrigation treatment, three crop loads were adjusted at bloom (high—100% natural crop load; medium—66%; low—33%).

Crop water requirements under drip irrigation were calculated using the following equation: ET_C_ = ET_0_ × K_c_ × K_r_. The reference evapotranspiration (ET_0_) was calculated using the FAO-Penman–Monteith equation [42], where K_c_ is a crop coefficient for sweet cherry reported by Marsal [43] and K_r_ is a localization factor [44] related to the percentage of ground covered by the crop.

The experimental design was completely randomized in a 2 × 3 factorial scheme (irrigation × crop load level) with three replicates per treatment (three trees per treatment).

### 4.2. Soil, Tree, and Fruit Water Status Measurements

The plant water status was measured weekly by measuring midday stem water potential (Ψ_stem_) at solar noon following the methodology proposed by McCutchan and Shackel [45] with a Scholander pressure chamber (Model 3000, Soil Moisture Equipment, CA, USA) in healthy and mature leaves located close to the trunk, two leaves per tree, six leaves per treatment. The fruit water potential (Ψ_fruit_) was measured at three fruit stages (green, straw, and maturity) on cut slices of the pitless fruits, six fruit per treatment, using a WP4C Dewpoint Potentiometer (Decagon Devices, Inc., Pullman, WA, USA) following the procedure of Léchaudel [46]. Fruit osmotic potential, flesh, and skin, (Ψ_ᴫf_) were measured in the same picked fruit used to measure Ψ_fruit_ using a vapor pressure osmometer (Wescor Vapro 5600, Logan, UT, USA) for the same three stages for the flesh (but measuring only the maturity stage for the skin). The estimated fruit turgor potential (Ψ_ρ_) was estimated as the difference between osmotic and fruit water potential according to Milad and Shackel [47].

The stomatal conductance (g_s_) and net assimilation rate (P_n_) were measured at solar midday every 7 days using a portable photosynthesis system CIRAS-2 (PP System, Amesbury, MA, USA) with a PLC6(U) cuvette at a photosynthetic photon flux density (PPFD) ≈ 1500 μmol m^−2^ s^−1^; leaf temperature was held at 25 °C and CO_2_ reference concentration at 380 μmol mol^−1^ on two sun-exposed leaves per tree, six leaves per treatment, on the same three trees per treatment that were used for the Ψ_stem_ measurement.

Branch diameter fluctuations were recorded using three dendrometers (LVDT sensors, model DF ± 2.5 mm, accuracy ± 10 μm, Solartron Metrology, Bognor Regis, UK) per treatment, each on a main tree branch away from direct sunlight. The sensors were installed on aluminum and invar holders to prevent thermal expansion. The maximum daily branch shrinkage (MDS) was calculated as the daily difference between the maximum and the minimum branch diameter. The soil matric potential (Ψ_m_) was measured by means of two thermal compensation capacitive sensors per replicate (MPS-6, Decagon Devices, Inc., Pullman, WA, USA) at 0.25 and 0.50 m depth and at a distance of 0.23 m from the emitter and 1.5 m from the trunk, under the canopy projection. Continuous measurements of branch diameter fluctuations and matric potential were recorded every 30 s, and the datalogger was programmed to report the means every 10 min (Campbell Scientific, Logan, UT, USA). Daily agrometeorological data were recorded by a weather station near the experimental orchard owned by the Spanish Agroclimatic Information Service (SIAR; http://crea.uclm.es/siar/datmeteo/, accessed on 7 August 2019).

### 4.3. Fruit Yield and Quality

At harvest, all the fruit produced per tree were harvested from the three trees considered per treatment and weighed in order to obtain fruit yield. To estimate fruit load, the weight of 100 fruits per tree was registered on 18 trees, 9 trees per irrigation treatment (CTL and DI), and 6 trees per crop load (high, medium, and low), to determine fruit unitary weight; consequently, fruit load (number of fruits per tree) was calculated from the yield (kg tree^−1^) and fruit unitary weight (g) measured in the field. Similarly, the fruit efficiency (FE) was calculated as the number of fruits per cm^2^ of the trunk cross sectional area (TCSA), and the water productivity (WP) was calculated as the kilograms of fruit produced by each tree per m^3^ of water applied. In order to evaluate the thinning and irrigation effects on fruit quality at harvest, 40 representative sweet cherries were picked per replicate (tree), 120 fruits per treatment. Of those, 20 fruits were used for the quality determinations and the other 20 were used to assess the cracking incidence. The quality parameters studied were equatorial diameter, fresh and dry weight, fruit color, soluble solids concentration, and titratable acidity. The equatorial diameter (mm) was measured with a digital caliper (model 17-262, Acha, Eibar, Spain). Fresh unitary mass (g) was estimated as the average of the unitary mass of 10 fruits from the same replicate weighed on an electronic balance (model AX623, Sartorius, Gottingen, Germany). Dry mass (g) was measured from the same fruits, which were dried at 60 °C until they were at a constant weight in a ventilated oven (model Digitheat, JP Selecta, Barcelona, Spain). Relative water content (RWC, %) was calculated by subtracting the dry weight from the fresh weight and referring it to the fresh weight. Fruit color was recorded using a colorimeter (CR-400, Minolta, Tokyo, Japan). Lightness and hue angle (hue°) were obtained from the L*, a*, and b* values of the CIE Lab scale system used. The soluble solids concentration (SSC, %) was determined with a digital hand−held refractometer (model N1, Atago, Tokyo, Japan) at 20 °C from the juice of the remaining 10 fruits per replicate, obtained with a hand press squeezer. Titratable acidity (TA, %) was measured with a titration (model 716 DMS Titrino, Metrohm, Herisau, Switzerland) and calculated from the volume of NaOH (0.1 M) needed to reach a pH of 8.1.

With the aim of assessing whether the interaction between irrigation and crop load management can affect fruit susceptibility to crack, the cracking index was measured in 20 fruits per replicate and 3 replicates following the procedure described by Christensen [48]. Cherries were immersed in 2 L distilled water (pH 7) at 20 °C, and crack presence on the fruit was evaluated after 2, 4, and 6 h. At each instance, cracked cherries were removed and recorded. The cracking index was calculated as:

Cracking index = 100 × [5a + 3b + c] × (5N)^−1^ (1)

In this Equation (1), a, b, and c represent the number of cracked fruits at 2, 4, and 6 h of immersion, respectively, and N is the total number of fruits measured (N = 20).

### 4.4. Statistical Analysis

At harvest, analysis of variance (ANOVA) and multivariate analysis of variance (MANOVA) were carried out to determine significant (*p* = 0.05) differences between the treatments. The degree of agreement between the independent variables (crop load) and the dependent variables (fruit cracked at harvest, cracking index, osmotic potential) was assessed using regression analysis. Statistical analyses were carried out using IBM SPSS Statistics v24 (Armonk, NY, USA). Principal component analysis (PCA) was carried out in RStudio package (RStudio Inc., Boston, MA, USA).

## 5. Conclusions

This study suggests that irrigation and crop load management affect sweet cherry tree and fruit water relations, fruit quality, and susceptibility to rain-induced fruit cracking. The cherries from trees submitted to deficit irrigation resulted in higher soluble solids concentration, lower fruit and osmotic potential of the flesh and skin at harvest, lower turgor potential, and lower unitary mass. On the other hand, the combination of high fruit turgor potential and a large gradient between the skin and flesh osmotic potentials in cherries from the CTL treatment resulted in a higher incidence of rain-induced cracking at harvest. The crop load also influenced the cracking incidence as well as the fruit quality. Thus, cherries from trees with low crop loads resulted in large size and high turgor potential and were more prompt to crack than those from trees with medium and high crop loads.

## Figures and Tables

**Table 1:** Yield, number of fruits per tree, fruit efficiency (FE), double fruit, cracked fruit at harvest, cracking index, and water productivity (WP) of ‘Prime Giant’ sweet cherries under two irrigation treatments, control (CTL), and deficit irrigation (DI) and three levels of crop load (high—100%, medium—66%, low—33%).

	Yield (kg Tree^−1^)	n Fruit(n Fruit Tree^−1^)	FE (n Fruit cm^−2^ Trunk)	Double Fruit (%)	Cracked Fruit (%)	Cracking Index	WP (kg m^−3^)
CTL33	8.13 b	599 b	2.35 b	0.17	31.50	63.47	2.67 b
CTL66	18.19 a	1401 ab	5.34 ab	0.17	28.50	59.73	6.07 a
CTL100	25.31 a	1944 a	7.04 a	0.50	21.33	60.00	8.44 a
ANOVA	0.005	0.006	0.025	0.492	0.412	0.153	0.004
DI33	8.91 b	680 b	2.40 b	1.00	10.33	53.87	4.75 b
DI66	20.06 ab	1694 a	6.09 ab	0.83	4.83	50.67	10.70 ab
DI100	27.52 a	2351 a	9.28 a	1.33	9.83	49.07	14.68 a
ANOVA	0.007	0.004	0.019	0.911	0.354	0.092	0.007
CTL	17.21	1304	4.91	0.28	27.11	61.07	5.73
DI	18.83	1598	5.92	1.06	8.33	51.20	10.05
ANOVA	0.695	0.412	0.550	0.094	0.001	0.001	0.033

Each value is the mean of the three replicates. Different letters on the same parameter (column) denote significant differences among levels of crop load within the same irrigation treatment, according to Duncan’s multiple range test (*p* < 0.05). In the ANOVA row, the *p*-values of each parameter are included.

**Table 2:** Effect of the irrigation treatment (control (CTL) and deficit irrigation (DI)) and crop load level (high—100%, medium—66%, low—33%) on fruit quality characteristics of ‘Prime Giant’ sweet cherries.

	Equatorial Diameter (mm)	Fresh Mass (g)	Dry Mass (g)	Relative Water Content (%)	Skin Color (hue°)	Soluble Solids Concentration (%)	Titratable Acidity (%)
CTL33	31.73 a	13.40	3.14	77.59	17.05	21.57	0.99
CTL66	31.48 a	13.01	2.94	78.43	21.98	20.50	0.94
CTL100	30.48 b	13.06	2.75	78.79	19.78	18.93	0.91
ANOVA	0.035	0.239	0.297	0.760	0.226	0.253	0.140
DI33	31.05 a	13.14 a	3.17	76.21	10.33 b	24.83	1.19 a
DI66	29.32 b	11.82 b	2.75	76.26	14.17 ab	22.97	1.08 ab
DI100	29.15 b	11.70 b	3.03	74.05	16.60 a	22.30	0.99 b
ANOVA	0.014	0.016	0.482	0.706	0.047	0.089	0.045
CTL	31.23	13.17	2.94	78.27	19.60	20.33	0.94
DI	29.84	12.21	2.98	75.51	13.70	23.37	1.09
ANOVA	0.004	0.004	0.825	0.043	0.002	0.002	0.003

Each value is the mean of the three replicates. Different letters on the same parameter (column) denote significant differences among levels of crop load within the same irrigation treatment, according to Duncan’s multiple range test (*p* < 0.05). In the ANOVA row, the *p*-values of each parameter are included.
